# Plague and the Human Flea, Tanzania

**DOI:** 10.3201/eid1305.061084

**Published:** 2007-05

**Authors:** Anne Laudisoit, Herwig Leirs, Rhodes H. Makundi, Stefan Van Dongen, Stephen Davis, Simon Neerinckx, Jozef Deckers, Roland Libois

**Affiliations:** * University of Antwerp, Antwerp, Belgium; † University of Liège, Liège (Sart Tilman), Belgium; ‡ University of Aarhus, Kongens Lyngby, Denmark; § Sokoine University of Agriculture, Morogoro, Tanzania; ¶ Katholieke Universiteit Leuven, Leuven, Belgium

**Keywords:** Plague, Yersinia pestis, fleas, Tanzania, research

## Abstract

Domestic fleas were collected in 12 villages in the western Usambara Mountains in Tanzania. Of these, 7 are considered villages with high plague frequency, where human plague was recorded during at least 6 of the 17 plague seasons between 1986 and 2004. In the remaining 5 villages with low plague frequency, plague was either rare or unrecorded. *Pulex irritans*, known as the human flea, was the predominant flea species (72.4%) in houses. The density of *P. irritans,* but not of other domestic fleas, was significantly higher in villages with a higher plague frequency or incidence. Moreover, the *P. irritans* index was strongly positively correlated with plague frequency and with the logarithmically transformed plague incidence. These observations suggest that in Lushoto District human fleas may play a role in plague epidemiology. These findings are of immediate public health relevance because they provide an indicator that can be surveyed to assess the risk for plague.

## Abstract 2

*Pulex irritans* fleas were more common in villages with high plague incidence.

Plague, caused by infection with *Yersinia pestis*, persists in many parts of the world; several hundred cases are reported to the World Health Organization each year, mostly from Africa (*1*,*2*). In Tanzania, a persistent focus of human plague was discovered in 1980 in the Lushoto District, in the northeastern part of the country. By 2004, 7,603 cases had been reported from this region (*3*). The distribution of plague cases in Lushoto is limited to an area of ≈1,200 km^2^, and a strong variation in plague frequency and incidence is seen among the villages in this region (*3*). Although evidence of infection with *Y. pestis* has been observed in several wild rodent and flea species, the actual reservoir in which the infection survives between epidemics has not yet been identified, and the ecology of the infection and the source from which humans acquire infection are poorly understood (*4*–*8*). In Lushoto District, frequent plague outbreaks occur in some villages, but the disease is uncommon in other villages in the same vicinity. A study is under way to compare the ecologic conditions in villages having frequent outbreaks with those in villages where plague is relatively rare, with the objectives of understanding, predicting, and ultimately controlling human plague. Comparing host and vector communities is an important part of such studies.

In Lushoto District, it has been suggested that the fleas *Xenopsylla cheopis*, *X. brasiliensis*, and *Dinopsyllus lypusus* are plague vectors among sylvatic rodents, but *Pulex irritans*, the human flea, has received little attention (*9*). *P. irritans* has been collected in several plague-affected and plague-free villages of the Lushoto area during epidemics and interepidemics (*10*), as well as on *Rattus rattus* (B.S. Kilonzo and S. Msingwa, unpub. data). Plastering a mud house is recommended in the area as a way of keeping the house free of fleas (*9*) and involves mixing soil (without manure) with water and rubbing the mixture over the floors with a piece of cloth. We report differences between plague-affected and plague-free villages in the numbers of free domestic fleas present in mud houses and consider whether this variation can be linked to house plastering as an antiflea measure.

## Materials and Methods

Lushoto District is situated in Tanga region, in the West Usambara Mountains, a part of the Eastern Arc Mountains. With an elevation ranging from 900 to 2,250 m above sea level, Lushoto District (04°22′–05°08′S, 038°05′–038°38′E) covers a surface area of 3,500 km^2^, of which 2,000 km^2^ are arable land and 340 km^2^ are forest reserve. Soils are mainly low-pH loams, rich in iron, manganese, and magnesium. Agriculture is the major economic activity, on which >90% of the population depends (*11*,*12*). The temperate climate is characterized by a short rainy season during November–December and a longer one during March–May. A minor and unreliable rain, the Mlwati, occasionally occurs in August and September. The region is the most densely populated area in Tanzania, with an annual growth rate of 2.8% and 102 inhabitants per square kilometer. Inhabitants belong to 1 of 3 major tribes: Wasambaa (80%), Wambugu (10%), and Wapare (5%); the remaining 5% are immigrants from diverse other regions (*13*) Most Lushoto residents (70%) keep livestock in their houses, but cats and dogs are usually kept outside (*9*).

**Figure 1:**
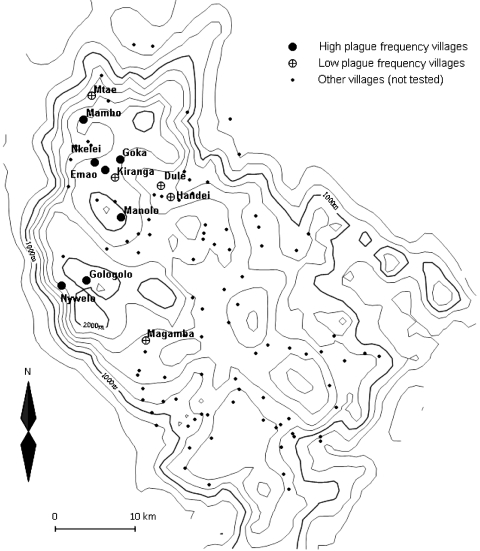
Map of the Lushoto District of Tanzania showing locations of villages with high and low plague frequency villages. All other villages with known locations are also plotted. The solid lines represent altitude contours (200-m elevation lines). To the west, a steep escarpment demarcates the edge of the district.

We selected 12 villages from throughout the plague-endemic area, ensuring a variation in both the frequency and incidence of plague, based on the earlier study by Davis et al. (*3*). Plague frequency is expressed as the percentage of plague seasons from 1986 to 2004 with reported plague cases, while plague incidence is the mean annual number of plague cases per 1,000 inhabitants at village level for the same period. Table 1 lists the 12 villages we surveyed, ranked according to plague frequency, and Figure 1 shows a map of the study area in Lushoto District. The 7 villages where plague on average occurred in >3 years per decade were considered “high plague frequency” villages, and the 5 villages where it occurred on average in <2 years per decade were considered “low plague frequency” villages. In all 12 villages, the common housing is mud houses with dirt floors and iron sheet or thatch roofs. Cattle are kept outside the house attached to poles during the day and feed on grass, but they are kept inside overnight.

**Table 1:** Data on villages in Lushoto District, Tanzania, surveyed for domestic fleas, ranked by plague frequency*

Village	Population†	Plague frequency	Plague incidence	Coordinates, South–East	Altitude, m asl	Year of most recent plague case	Year(s) flea trapping conducted	No. forms received‡
Dule	3,036	0.059	0.637	04.58370–038.31657	1,405	1986	2006	20
Mtae	3,407	0.059	0.121	04.48421–038.23758	1,632	2000	2006	20
Handei	5,745	0.118	0.137	04.59514–038.32390	1,376	1990	2006	20
Kiranga	868	0.176	1.056	04.57571–038.27021	1,821	1996	2005–06	33
Magamba	2,676	0.176	0.836	04.72895–038.30148	1,743	1998	2005–06	52
Goka	1,116	0.353	1.175	04.56680–038.25990	1,843	1997	2006	20
Mambo	5,669	0.353	0.722	04.51167–038.21976	1,828	1997	2006	20
Nkelei	1,305	0.647	3.434	04.56062–038.24209	1,904	2000	2006	20
Shume-Nywelo	3,757	0.647	10.460	04.70025–038.19687	1,890	2001	2006	20
Emao	2,054	0.706	5.180	04.56276–038.25304	1,827	2000	2005–06	41
Gologolo	2,202	0.765	18.544	04.69707–038.22692	1,950	2002	2005–06	26
Manolo	10,464	0.765	6.320	04.62058–038.22260	1,809	2003	2006	9

*Data about plague are extracted from (*3*). Villages with a plague frequency of >0.3 on average, ≥3 years in a decade, were considered high frequency villages. asl, above sea level. †From 2002 census. ‡No. questionnaire responses received from village during survey.

Collection of domestic fleas began in May 2005 because earlier literature reported that plague cases in Tanzania usually appear 2 times per year, in October/November and May/June (*14*). A more recent detailed investigation of Lushoto hospital data, however, showed a consistent seasonal pattern in which the highest number of plague cases occurs in January (*3*). Taking into account the practical limitations of extended fieldwork periods overseas, a second collection period was started in January 2006. Fleas were thus collected every month from May through August 2005 in 4 core study villages (Gologolo, Emao, Kiranga, and Magamba) and from January through March 2006 in all 12 villages.

Houses surveyed were randomly chosen after the chief of each village granted authorization. Fleas were trapped by using a kerosene hurricane lamp hung above a 3-cm–high tray with a 45-cm diameter, half full of water. The lamp was lit at dusk and switched off at dawn during 3 consecutive nights. All traps were checked every morning between 9 am and 11 am, and captured fleas were preserved in 70% ethanol. The head of each household was interviewed by questionnaire to assess the perception of flea nuisance in the house and the frequency of plastering.

We calculated the *P. irritans* index (*Pii)*, per village per month, as the average number of *P. irritans* collected in a house. Frequency and incidence are village-specific characteristics based on long-term data, while for *Pii* we had to rely on relatively sparse and heterogeneous sampling during a short period. Simple averaging and testing for a correlation may be misleading if findings vary between days, months, or years. Therefore, we analyzed the relation with a mixed model that included these sources of temporal variation in *Pii* (first applying a log transformation to ensure normality). This model included log(*Pii*) as the dependent variable and frequency or log(incidence) as the (continuous) independent factor. Year and month (nested within year) were added as fixed effects, while village and the year–village interaction were treated as random effects to control for temporal variation in *Pii*, as well as for the fact that village is the independent unit of observation. Correlations in day-to-day estimates of *Pii* were modeled by using an autoregressive correlation coefficient. Model selection was based on backward elimination of nonsignificant fixed effects. All random effects were retained in the model to ensure appropriate weighting and approximation of degrees of freedom by using the Kenward-Roger method.

We also tested for lagged relationships between monthly values of *Pii* and monthly plague incidence, that is, monthly incidence; was related to the mean *Pii* for the previous month. Per village, the average number of people infected with plague each month was further expressed as a proportion of total number of cases in the district and was termed the monthly incidence. Daily flea numbers per house per village were summed to obtain a mean monthly *Pii* ± SD. The natural logarithm of the monthly incidence +1 was the dependent variable, while the natural logarithm of the mean monthly *Pii* was the predictor variable. Possible temporal dependence of the monthly incidences within villages was modeled by using an exponential decay of the degree of temporal autocorrelation. We also tested for an association between plastering frequency (independent variable) and the total number of captured fleas (dependent variable, log transformed) in a mixed analysis of covariance model with village and village-by-frequency as random effects. In all the analyses above, standard error and denominator degrees of freedom were estimated by the Kenward-Roger method. We also tested for an association between altitude and the mean monthly *Pii* (log transformed to ensure normality) by using a linear regression model. Finally, the association between flea abundance and the time since the previous plastering (the number of days between the last time the householder said the floor was plastered and the date of our first visit) was analyzed with a Cox proportional hazards model. If no fleas were trapped, the observation was considered to be censored. All analyses were performed in SAS version 9 (SAS Institute Inc., Cary, NC, USA).

## Results

*P. irritans* was the predominant species (72.4%) among domestic fleas. Other species collected were *Echidnophaga gallinacea* (15.1%), *Ctenocephalides felis* and *C. canis* (6.5%), *Xenopsylla brasiliensis* (3.4%), and *Tunga penetrans* (2.6%). *P. irritans* and *E. gallinacea* were the only species found in every village. *P. irritans* accounted for 61.5% and 75.2% of all fleas collected in low and high plague frequency villages, respectively (Table 2). Twice as many houses were infested by *P. irritans* in high plague frequency villages than in low plague frequency villages (Table 2).

**Table 2:** Distribution of flea species within villages and houses, Lushoto District, Tanzania

Domestic flea species	Common hosts in Tanzania	Flea species composition, %		Houses with given flea species, %	
		Low*	High†	Low*	High†
*Pulex irritans*	Humans	61.5	75.2	28.8	65.4
*Ctenocephalides felis*, *C. canis*	Cats, dogs, other animals	8.8	5.8	6.8	10.7
*Echidnophaga gallinacea*	Domestic fowl, *Rattus rattus*	19.6	13.7	12.2	15.7
*Tunga penetrans*	Humans, dogs, goats	2.0	2.8	2.0	6.9
*Xenopsylla brasiliensis*	*Rattus rattus, Mastomys natalensis*	6.8	2.1	4.1	5.0

*Villages designated as low plague frequency. †Villages designated as high plague frequency.

**Figure 2:**
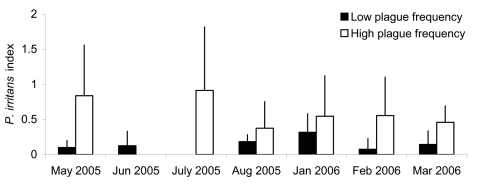
Monthly domestic *Pulex* *irritans* index, averaged for low plague frequency villages (black columns) and high plague frequency villages (white columns). The error bars indicate standard deviation from the mean. No data were available for high plague frequency villages in June 2005 or for low plague frequency villages in July 2005.

For all the trapping sessions in 2005 and 2006, the *P. irritans* index was 2−9× greater in high plague frequency than in low plague frequency villages (Figure 2). The statistical analysis also showed that *Pii* was strongly positively correlated with plague frequency (F_1,11.3_ = 14.08, p = 0.003), with the logarithmically transformed plague incidence (F_1,11.5_ = 12.62, p = 0.004) and altitude (F_1,11H_ = 8,641, p = 0.015). The abundance of other species was much lower than that of *P. irritans*, and none of the other flea species indexes were correlated with plague frequency or incidence.

The questionnaires (301 valid responses) showed that in 8 of the 12 studied villages, some persons plaster their houses daily (Table 3). The figures suggest a great variability in the frequency of plastering between and within villages and that frequency of plastering has no relation with the frequency of plague. For example, in Shume-Nywelo (high plague frequency) and Dule (low plague frequency), 55% and 50% of housekeepers, respectively, said they never plaster the house; in Gologolo (high plague frequency), 65.4% plaster their houses 7 times a week, but in Emao (another village with high plague frequency), only 19.5% do so.

**Table 3:** Questionnaire responses about plastering frequency, Lushoto District, Tanzania

How many times per week do you plaster the floor?								
Village	0	1	2	3	4	5	7	n*
Low frequency								
Dule	10	–	1	1	7	1	–	20
Mtae	9	–	–	–	2	1	8	20
Handei	–	1	–	–	–	–	19	20
Kiranga	13	1	8	5	4	–	2	33
Magamba	24	6	3	8	4	1	6	52
High frequency								
Goka	–	1	–	–	–	–	19	20
Mambo	5	–	–	2	3	–	10	20
Nkelei	6	–	–	–	1	–	13	20
Shume	11	–	3	3	3	–	–	20
Emao	26	–	–	4	1	2	8	41
Gologolo	9	–	–	–	–	–	17	26
Manolo	5	–	–	–	1	–	3	9
Total	118	9	15	23	26	5	105	301

*No. respondents per village.

The frequency of plastering did not correlate with the natural logarithm of the total number of fleas caught (*t*_20_ = 0.88, p = 0.39), and this lack of association did not vary across villages (no significant random village–frequency interaction, χ^2^_1_ = 0.4, p = 0.47). Frequent plastering did not appear to prolong the time between the last plastering and the occurrence of the first fleas in the trap (χ^2^_1_ = 0.36, p = 0.55).

## Discussion

Our results show that the density of domestic fleas is higher in villages with a higher plague frequency or incidence. Moreover, the human flea *P. irritans* accounts for a larger percentage of the domestic fleas in these villages. The factors that contribute to the presence of plague in some villages in Lushoto while it is absent from others (*3*) are so far unknown. It is tempting therefore to attribute an epidemiologic role to *P. irritans*. This has been suggested recently for another focus of human plague; Arrieta et al. (*15*), working in the Peruvian Andes, observed that 69.9% of fleas collected in domestic environments (on domestic animals and inside houses) were *P. irritans* (or, perhaps, *P. simulans*, a sister species) and found the same positive relation between high plague risk areas and *P. irritans* densities.

The human flea was first mentioned in tropical Africa (Ethiopia) in 1868 (*16*). In Tanzania, plague was first reported in 1886 in the Iringa region, but no information is available about the flea species present at that time. The presence of *P. irritans* in Tanzania dates at least to 1915, when it was found in Dar-es-Salaam. In northeastern Tanzania, its presence was reported in 35% of the beds examined by Smith in 1959 (*17*); in 1977, 82.5% of the fleas collected in human dwellings belonged to this species (*18*). *P. irritans* is often found in high densities in habitations, especially those with a dirt floor and a thatched roof, and is considered a possible plague vector in Angola, Brazil, Burundi, Democratic Republic of Congo, Iran, Iraq, Nepal, People’s Republic of China, and Tanzania (*19*–*21*).

Although a substantial body of literature describes the ecology of plague, the relation between the bacterium *Y. pestis* and the human flea *P. irritans* during epizootics and epidemics is poorly understood. The classic epidemiologic model for plague considers it an enzootic infection of mostly resistant wild rodents. An outbreak of human plague may begin with an epizootic in peridomestic rats, from which rodent fleas (in tropical regions typically *X. cheopis*) questing for a host may infect humans (*22*). In this scenario, human ectoparasites do not play an important role. However, epidemiologic investigations based on historical accounts of the Black Death in the 14th–16th centuries in Europe show that the epidemics do not conform to this classic model, even leading to suggestions that the Black Death may have had a cause other than *Y. pestis* plague, an issue that is still hotly debated among historians (*23**,**24*). Recently, Drancourt et al. (*25*) reviewed earlier biologic studies that have presented experimental evidence for or against the role of *P. irritans* in the transmission of plague.

*P. irritans* is frequently infected with *Y. pestis* (pestiferous) but is rarely infective (China, Ecuador, Kazakhstan, Democratic Republic of Congo, Brazil; [*21*]), mainly because it is not an easily blocked species (*21*). Blocking of the proventriculus by massive replication of the *Y. pestis* bacteria is known to enhance flea vectorial capacity and occurs in known plague vectors *X. cheopis* and *Nosopsyllus fasciatus* (*26*). Therefore, the role for *Pulex* spp. as plagues vector was classically believed to be no more than mechanical transmission by way of soiled mouthparts, which is only possible if a high level of bacteremia exists in the pestilent host, if new potential hosts are available within 3 days after the infective blood meal, and if multiple bites occur (*21*). Such levels of ectoparasitism are realistic in a rural habitat; for example, in 1 night in our study in Gologolo, a basic light trap caught 26 fleas in a single room.

The role of unblocked fleas may, however, be more than just mechanical. Eisen et al. (*27*), studying alternative fleaborne transmission mechanisms, recently showed that *Oropsylla montana*, which rarely becomes blocked, is immediately infectious, transmits efficiently for at least 4 days postinfection (early phase), and may remain infectious for up to 8 weeks postinfection because the fleas do not undergo block-induced death. This scenario of efficient early-phase transmission by unblocked fleas matches historical observations of rapidly spreading epizootics and epidemics and their highly focal nature. During the second plague pandemic, in Europe, *P. irritans* was a suitable vector because it was abundant on persons and in their homes, as it is today in some remote foci in Central Asia (*25*,*28*). In Ecuador, during a plague outbreak in the Chimborazo region in 1998, *P. irritans* was abundant in human bedding (*29*). The findings of the study by Eisen et al. (*27*) would also be consistent with a role for human fleas in the epidemiology of plague in Lushoto. In contrast, in the Ituri plague focus in the Democratic Republic of Congo, Devignat noticed the total absence of domestic *P. irritans* (*16**,**30*), just as in the epidemics in Saigon-Cholon in 1943 (*31*). *P. irritans* also appeared later in foci in the Democratic Republic of Congo, and the primary human fleas at that time (1946) were *X. cheopis* and *X. brasiliensis* (*32*).

Among the other domestic species collected, *C. felis strongylus* and *C. canis* are commonly found on cats and dogs in Lushoto (*5*). These species are poor plague vectors but can be pestiferous, as observed in Democratic Republic of Congo (*30*). *T. penetrans*’ status as plague vector is unknown. The females of this species are embedded in the host epidermis (humans, dog, rat, cat), but males are free hematophagous ectoparasites (*33*). *E. gallinacea* is frequent in human homes where hens are kept, but it was never observed on humans in Lushoto. It has been found to be infected with *Y. pestis* in the field (*34*,*35*) but is considered a poor plague vector due to its “stick tight” behavior (*36*). Finally, *X. brasiliensis* is the African counterpart to Asian *X. cheopis* in the sense that it is considered an excellent plague vector (*7*,*30*). Notably, the abundance of *X. brasiliensis* could not explain the village-level variation in either incidence or frequency of human plague in the present study.

During our study, no human plague cases were recorded in the test region, and the small mammals we trapped in the 4 core villages tested negative for *Y. pestis* (n = 925, tested in a multiplex PCR; data not shown). Thus, the study period could be atypical in the sense that it is a period in which plague was absent. Whatever the explanation for the absence of plague cases, it is nevertheless clear that the abundance of *P. irritans* differs significantly between villages with different histories of human plague cases.

Because the vectorial status of *P. irritans* is still under discussion, and because of the correlative nature of our results, the observed relations must be interpreted with care. For example, *P. irritans* may not be a significant plague vector but a biologic indicator of the conditions that are conducive for the occurrence of plague in a village. Flea larvae are very sensitive to moisture excess and dehydration, 2 conditions that are caused by abiotic factors, mainly air/soil humidity and temperature, factors likely to vary locally and annually. Climatic conditions are further linked with altitude and orientation of slopes in mountainous areas, and those do not change from 1 year to another. Indeed, elevation cannot change the transmission of plague, but it can create conditions that are more conducive for plague, such as the distribution of particular flea species. Altitude effects on the distributions of sylvatic flea species are partly explained by host availability and population density but also by local climatic conditions (*37*). For example, in the Madagascar highlands, at an altitude <800 m, the sylvatic flea *Synopsyllus fronquerniei* is absent, even though its common host, *R. rattus*, is present (*38*). Soil texture can also affect both development time and survival of preimaginal stages of fleas through differences in soil moisture (*39*).

Our data suggest that human fleas may play an important role in spreading plague in Lushoto, or that human fleas at least are correlated with other factors that are important in this respect. These observations are of immediate public health relevance because they provide a clear indicator that can be surveyed to assess plague risk. Also, they suggest a clear target to be included in disease control efforts and indicate where to continue looking for factors that are responsible for the persistence of plague foci. Earlier studies have so far not been able to pinpoint such factors in the Lushoto plague focus, nor in the similar focus of Okoro County, Nebbi District, Uganda, which has been surveyed for 13 years (*4*,^6^,*14*,*40*). Plague has always been associated with poor home and environmental sanitation, and plague control in Africa has always focused on rodents and their fleas. Our results show the importance of including human ectoparasites in control programs and that plastering of houses, a locally accepted means of flea (and plague) control, does not have the expected effect on flea densities.
